# Sustainable high-entropy materials?

**DOI:** 10.1126/sciadv.ads3926

**Published:** 2024-12-11

**Authors:** Liuliu Han, Wangzhong Mu, Shaolou Wei, Peter K. Liaw, Dierk Raabe

**Affiliations:** ^1^ Max Planck Institute for Sustainable Materials, Max-Planck-Straße 1, 40237 Düsseldorf, Germany.; ^2^ Engineering Materials, Department of Engineering Science and Mathematics, Luleå University of Technology, 97187 Luleå, Sweden.; ^3^ Department of Materials Science and Engineering, The University of Tennessee, Knoxville, TN 37996-2200, USA.

## Abstract

High-entropy materials (HEMs) show inspiring structural and functional properties due to their multi-elemental compositions. However, most HEMs are burdened by cost-, energy-, and carbon-intensive extraction, synthesis, and manufacturing protocols. Recycling and reusing HEMs are challenging because their design relies on high fractions of expensive and limited-supply elements in massive solid solutions. Therefore, we review the basic sustainability aspects of HEMs. Solutions include using feedstock with lower carbon and energy footprints, sustainable primary synthesis routes from minerals, attenuation of the equimolar alloying rule, and a preference for scrap and dumped waste for secondary and tertiary synthesis. The high solubility, compositional flexibility, and chemical robustness of HEMs offer pathways for using higher fractions of mixed and contaminated scrap and waste feedstocks, which are not admissible for synthesizing conventional materials. We also discuss thermodynamic and kinetic design strategies to reconcile good material properties with high impurity tolerance and variable compositions.

## Teaser

Metallurgical strategies for enhancing sustainability aspects of high-entropy materials are outlined.

## INTRODUCTION

The objective of this paper is to examine and elucidate metallurgical strategies for enhancing the sustainability of high-entropy materials (HEMs) (*1*–*3*). HEMs consist of multiple principal elements blended in high or near-equimolar ratios, leveraging the thermodynamic principle of enhancing configurational entropy as a key factor for stabilizing solid solutions across a wide compositional range (*4*). However, despite the theoretical potential for achieving solid solutions with high thermodynamic stability, many HEMs decompose into multiple phases rather than maintaining stable single-phase microstructures (*5*–*10*). This originally unintended trend toward phase decomposition has been harnessed as a material design option in this field for producing materials with useful microstructures and chemical defect-decorated states, all of which are accessible through adequate kinetic treatments (*11*–*14*). Consequently, extensive compositional mixing has emerged as a promising strategy for material design across a wide range of material classes (*15*–*17*).

Several HEMs with chemically concentrated solid solution matrices, particularly metallic alloys, have exhibited promising properties, such as high strength and toughness (*18*, *19*), good magnetic and electrical characteristics, excellent catalytic properties (*20*–*22*), invar response (*23*–*26*), and good resistance to hydrogen attack (*27*, *28*), corrosion (*29*, *30*), oxidation, and abrasive loads (*31*, *32*). However, the compositional subspaces of material variants with adequate mutual solubility that target extensively mixed solid solutions often rely on elements that raise substantial sustainability concerns.

In a few cases, the remarkable properties exhibited by some of the HEMs found so far can nonetheless justify the use of these elements for materials when used for critical applications where certain enhanced properties are essential. Thus, substantial gains in performance can potentially outweigh concerns associated with their constituent elements, high production costs, energy-intense synthesis and processing, as well as the likewise carbon-intensive extraction of the elements from their ores. This argument applies particularly for products or processes with highly safety- and energy-critical requirements, where improved HEMs can potentially leverage substantial advantages in efficiency, cleanliness, less toxic by-products, and overall improved sustainability of downstream products and processes. These cases are referred to as indirect sustainability gains (see Table 1) (*33*).

**Table 1.** Research topics and opportunities for indirect sustainability effects leveraged by multi-functional HEMs.

Targeted multi-functionality	Specific examples of multifunctional HEMs
Mechanically robust and chemically lean magnetic materials	Mechanically strong, yet soft magnetic HEMs designed to function under harsh service conditions, such as high and cyclic mechanical loads, high temperature, and corrosive attack.
	Compositionally lean RE free hard magnetic/magnetocaloric HEMs.
Mechanically and thermally stable electrical materials	Mechanically stable and compositionally lean thermoelectric HEMs for higher conversion efficiency and safer operation.
	High electrical resistivity HEMs with low-temperature coefficients of the resistance exposed to high-temperatures and mechanical loads.
Chemically active and mechanically robust materials	Compositionally lean and catalytically active HEMs (e.g., in the form of thin films, nanoparticles, and porous materials) with high chemical activity, stability, selectivity, and reversibility.
	High-entropy hydrides with low mass density and high storage capacity for hydrogen storage and low and tuneable release barriers.
	Bulk and low-dimensional biomedical HEMs with low mass density, corrosion resistance and antiviral surface oxides. Alloy variants with partially opposite features—such as targeted gradual dissolution and absorption inside the body—are also conceivable.
Chemically and thermally robust structural materials	Mechanically robust low thermal expansion HEMs with low mass density and good corrosion resistance.
	High-entropy bulk materials and coatings for extreme irradiation and corrosive environments.
	High-entropy shape memory materials tuned for large switchable and reversible strain values to couple thermal, mechanical, electric, and magnetic properties.
	Recycling-friendly mechanically strong and damage tolerant HEMs with high impurity tolerance.
	High-entropy binder material with good wettability, high-temperature stability, good oxidation resistance, corrosion resistance, and fracture toughness.

These improvements in long-term sustainability can in certain cases justify the use of environmentally harmful elements and production protocols, which need to be documented by corresponding quantitative life cycle assessments. Conceivable cases along these lines may emerge in applications for advanced magnetic (*34*, *35*), efficient caloric (*36*, *37*), thermally stable (*38*–*40*), and hydrogen-resistant (*27*, *41*) and robust catalytically active materials (*20*–*22*), as well for application scenarios where harsh environmental service conditions apply (*42*).

**Fig. 1.**
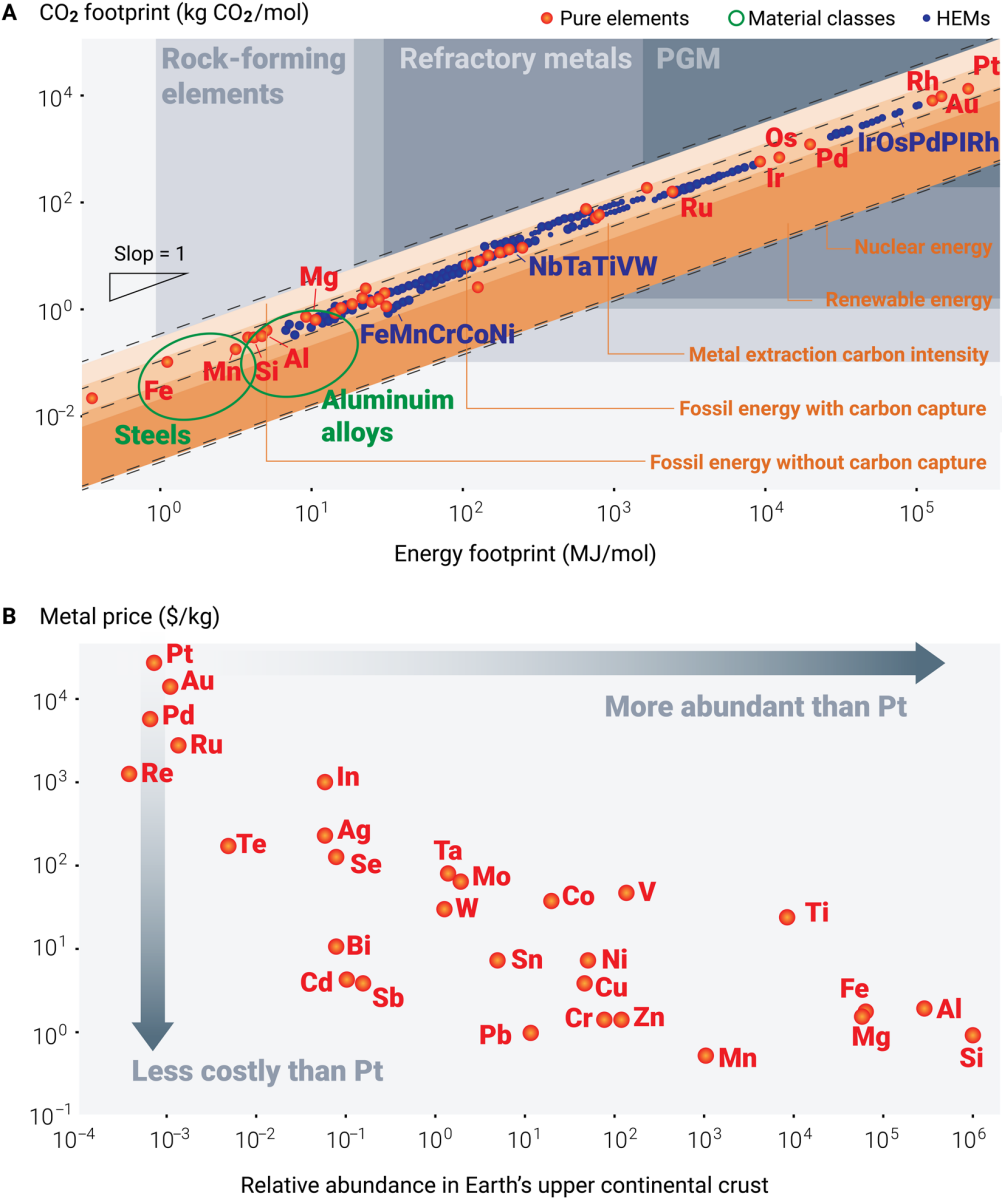
Energy, carbon, cost abundance considerations of HEMs and metals. (**A**) CO_2_ footprint associated with the energy required for the metal extraction of more than 30,000 different HEMs (blue dots). The values of pure metals (red dots) and larger material classes, such as steels and aluminum alloys (green circles), are also shown in (*155*). (**B**) Relationship between metal prices and the relative abundance of chemical elements in Earth’s upper continental crust on the basis of the abundance of silicon with 10^6^ atoms (*170*). Notably, not only the abundance but also the dispersion (richness) across a larger scale and within the ore minerals determines the CO_2_ and energy footprints of metal extraction. PGM, platinum group metal.

Three main groups of metallic HEMs with large solid solution regimes have been identified, on which most studies have been conducted so far. The first group includes face-centered cubic (fcc) structures based on the CoCrFeNiMn system (*1*–*3*), which uses elements well known from the domain of stainless steel design and has high mutual solubility in the fcc lattice. Elements such as nickel and cobalt are essential and present in high contents in this system, e.g., 20 atomic % (at %) for equimolar alloys and even higher for quaternary and ternary variants. However, these elements are expensive and strategically relevant, serving in high-price, low-volume applications such as battery electrodes, stainless steels, magnets, or superalloys and producing up to 30 times more CO_2_ emissions than steels per mass produced, as shown in Fig. 1A. The second group comprises body-centered cubic (bcc) structures that are based on alloy systems that use hexagonal elements such as titanium, zirconium, and hafnium mixed with bcc refractory elements such as niobium, tantalum, vanadium, molybdenum, and tungsten (*43*–*51*). Chromium and iron can also be used in this group. These massive solid solution systems are known for their high mutual solubility, which is known from bcc-structured beta titanium alloys or gum metals (*52*). Although this second group of HEMs is attractive because of its high mutual solubility, many of these elements are expensive, considered strategically critical, prone to oxygen and hydrogen uptake, and pose additional challenges in heat treatment and homogenization. These factors affect economic viability and industrial scalability because of the high associated energy consumption and CO_2_ footprint, which are typical features of most refractory metals. The third group includes hexagonal (*53*–*57*) and orthorhombic (*58*, *59*) crystal structure mixtures based on rare-earth (RE) elements such as yttrium, gadolinium, terbium, dysprosium, lutetium, or BeCoMgTi blends (*60*). Yet, many of these elements are among the most critical and expensive ones in use today, as they are needed in high-performance hard magnets, a material class of utmost relevance in the ongoing global transition toward electrification. In addition, some of them, such as beryllium, are environmentally hazardous or even toxic when extracted, processed, and used.

The trade-offs between the benefits arising from the enhanced properties of HEMs, on the one hand, and their associated environmental and economic shortcomings, on the other hand, necessitate addressing sustainability challenges for their responsible development and application (*61*–*67*). This study compiles the current sustainability metrics and examines the associated challenges of HEMs. Equipped with these results, we suggest research directions for developing more sustainable HEMs, focusing on both direct and indirect sustainability effects (*33*, *63*). Direct sustainability measures involve using minerals, scrap, or waste materials with lower CO_2_ and energy footprints in synthesis and processing, avoiding the use of critical elements and unfair labor conditions and considering the relative abundance of the chemical elements in Earth’s upper continental crust and their respective dispersion in minerals, scrap, and dumped waste (Fig. 1B) (*68*). Indirect sustainability measures pertain to the potential of these materials in leveraging more efficient and sustainable energy conversion applications, robust catalysis applications, lower corrosion rates, weight reduction, and resistance to hydrogen embrittlement, to name a few key features.

**Fig. 2.**
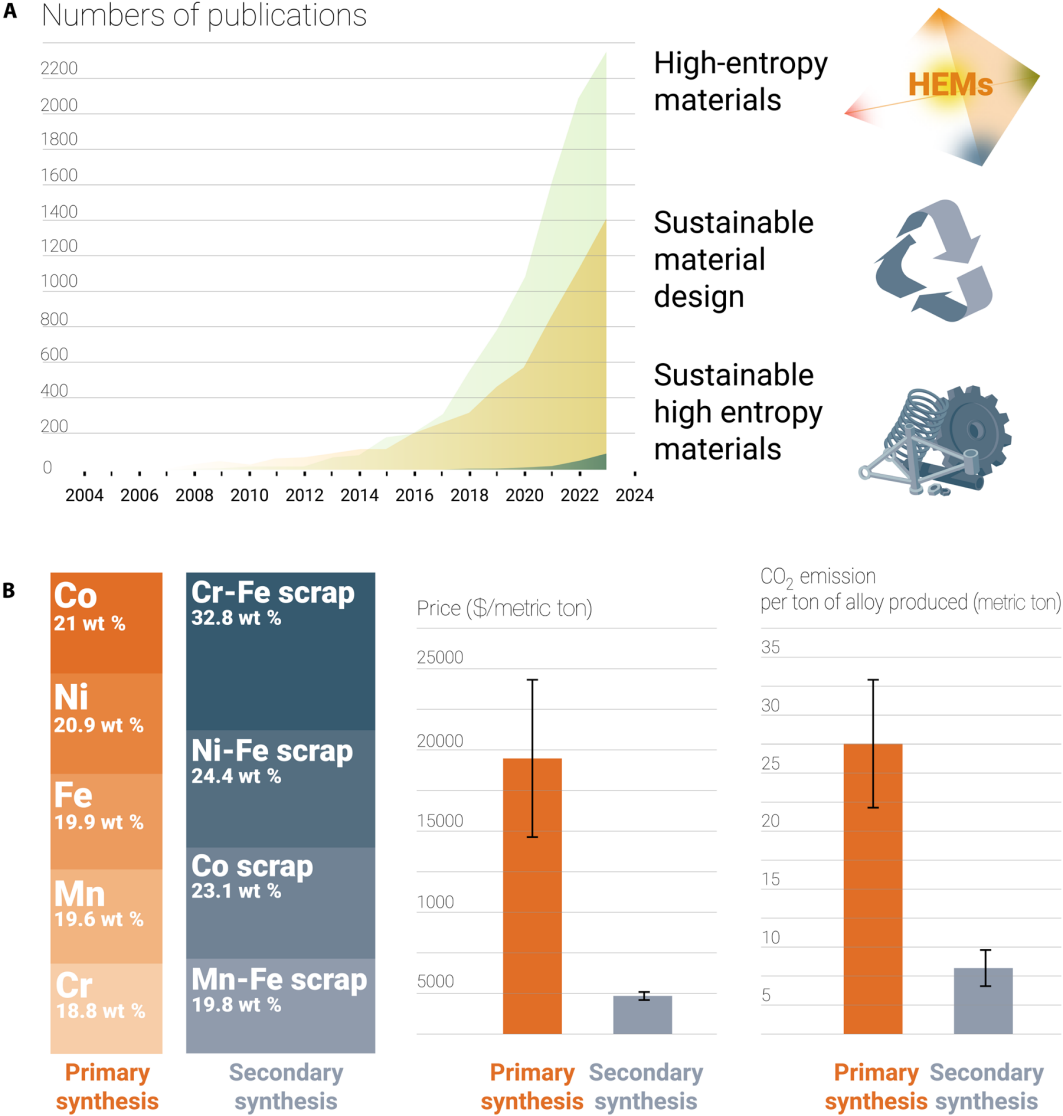
Increasing interest in sustainable high entropy materials design. (**A**) Increase in scientific literature output on HEMs, sustainable material design, and sustainable HEMs. (**B**) Comparison of some real-world key metrics considering the required raw materials for the CoCrFeNiMn (Cantor) alloy when fabricated via primary synthesis (high-purity metallic materials from minerals) and secondary synthesis (from scrap). The analysis shows the average values for the price and CO_2_ footprint.

The critical evaluation we target in this paper addresses the high-energy and high-CO_2_ footprints, as well as the complexity of recycling these materials owing to their multi-elemental chemical composition. The search for sustainable HEMs also aims to increase the cost efficiency of these materials, another unresolved downside of this material class. On the other hand, despite their high CO_2_ burden, some HEMs may, in the future, possibly compete with established materials that are even less sustainable. Examples include certain materials used today as catalysts, magnets, electrodes, or invar materials. These aspects underscore the need for research on the sustainable synthesis, application, and recycling of HEMs. However, the current knowledge gap is evident from the limited focus on sustainable HEMs, as clearly shown by screening the established literature on “HEMs,” “sustainable material design,” and “sustainable HEMs” (Fig. 2A).

With these data in mind, this study is therefore motivated by the aim of reviewing HEMs with a focus on metallurgical solutions to reduce their cost, energy, and carbon footprint during extraction, synthesis, manufacturing, application, and recycling. Potential solutions include (i) developing sustainable synthesis pathways; (ii) using untapped and more responsible feedstock sources from minerals (for primary synthesis, for metal extraction via ore beneficiation and reduction), scrap (secondary synthesis, i.e., circular use though adequate blending and remelting), and dumped waste (tertiary synthesis, where mixed contaminated and partially oxidized materials are used); (iii) using higher fractions of mixed and contaminated scrap and waste than conventional materials due to the often high solubility, compositional flexibility, and chemical robustness of the HEMs; and (iv) guiding all of the above by thermodynamic and kinetic design strategies and by artificial intelligence to identify the optimal match between feedstock and target HEMs to balance good material properties with high impurity tolerance.

### Direct sustainability aspects: Responsible synthesis and processing of HEMs

**Fig. 3.**
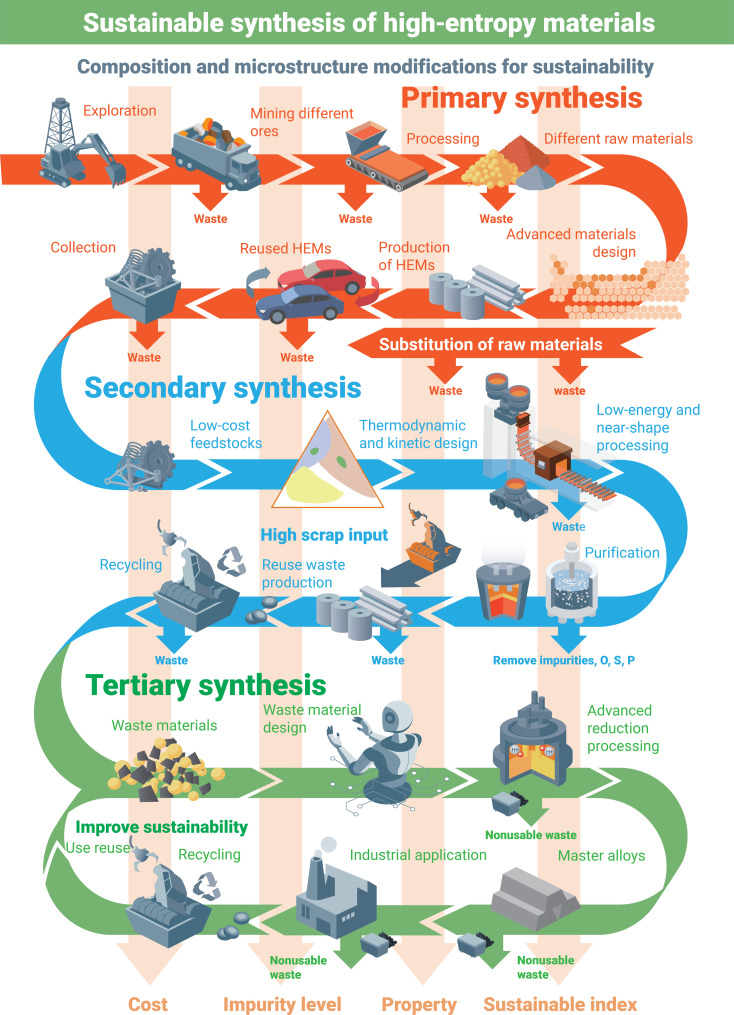
Future synthesis-related aspects of the metallurgical HEMs sector toward a more sustainable and circular economy. Reused materials, end-of-life products, and dumped waste all contribute to the manufacturing chain to achieve a more responsible carbon, energy, and scarcity footprint of these materials.

Methods for the production of HEMs can be classified into primary, secondary, and tertiary synthesis approaches, according to the use of different raw materials, i.e., high-purity metallic materials for primary synthesis, low-cost feedstocks for secondary synthesis, and dumped waste materials for tertiary synthesis, as shown conceptually in Fig. 3.

Conventional primary synthesis refers to the fabrication of HEMs from expensive and often environmentally harmful high-purity raw materials via established but usually less sustainable hydrometallurgical, electrometallurgical, and/or pyrometallurgical extraction, smelting, and blending processes (*69*). For instance, bulk nuggets, flakes, or powders of high purity, e.g., >99.5% (*70*), are extracted and purified from minerals and melted under a high purity protection atmosphere and subsequent metallurgical cleaning steps or high vacuum conditions to minimize the amount of impurities (*71*). To date, these steps constitute the main synthesis and cleaning methods used in most laboratory-scale HEM research. For example, many HEM systems have been synthesized and investigated using this method over the past two decades (*15*, *16*, *20*, *72*–*74*), e.g., Cantor-type CoCrFeNiMn HEMs (*1*, *2*), refractory Senkov-type TiZrHfNbTa HEMs (*43*), dual-phase eutectic HEMs (*75*), and transformation-induced plasticity (TRIP) effect HEMs (*12*).

**Fig. 4.**
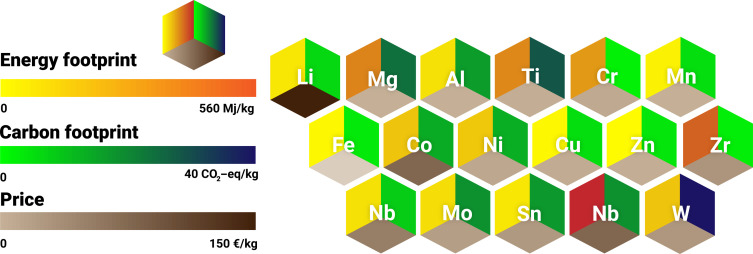
Sustainability and cost considerations for HEMs. Aspects such as sustainability, responsible use of elements, absolute price, and cost variations need to be considered when designing sustainable HEMs. Figure reproduced in modified form with permission from (*42*).

The good load-bearing performance of these materials—particularly strength, work hardening, ductility, and fracture toughness—is mainly due to their defect-based microstructure design, which combines different thermodynamic effects and kinetic mechanisms to equip HEMs with the required features (*76*). Mechanically promising alloys were, for example, developed by leveraging the metastability of the bulk phase to trigger twinning and phase transformation under mechanical loading. Some of these mechanisms are highly dependent on the precise control of the bulk composition, deformation rate, and temperature to gradually induce these athermal transformation effects when needed to move the Considère limit toward higher strains. For instance, the addition of a small amount of interstitial carbon (0.5 at %) into the non-equiatomic FeMnCoNi HEM can trigger a twinning-induced plasticity effect while maintaining the TRIP effect during tensile deformation by reducing the magnitude of the stacking fault energy and hence the phase’s stability against mechanical loading (*77*). In addition, the dissimilar solute redistribution (*78*) of the multiple principal alloying elements in HEMs affects the constitutional undercooling (*78*, *79*) ahead of the liquid-solid interface, a key factor influencing the dendrite growth characteristics and thereby the material’s propensity to hot crack (*80*–*82*). Therefore, the underlying compositional fine-tuning of HEMs typically requires metallic feedstock with high-purity levels as raw materials, followed by different alloy cleaning steps and, likewise, well-controlled metallurgical workflows to avoid vapor pressure losses and associated composition deviations and to suppress the introduction of impurities. In addition, compared with conventional metallic alloy synthesis routes, most of the abovementioned processes are less sustainable, cost-intensive, and energy-intensive, as indicated by the sustainability and cost considerations of using different elements for HEM design (Fig. 4), thus hindering their potential large-scale industrial application.

The two main HEM families that have been mostly studied to date and have shown promising properties are the Cantor and Senkov groups. These materials—as well as their many interstitially blended, nonstoichiometric, ternary, and quaternary variants and subgroups—face different specific sustainability challenges in primary and secondary synthesis. For the first HEM family, the Cantor alloys—particularly the production of metallic cobalt, nickel, and manganese—are extremely costly and CO_2_ intensive because these elements are very dilute in their natural ores, as outlined below. Primary metallurgical synthesis is, therefore, very costly and has an extremely high carbon footprint (Fig. 4). Also, some of these elements often come from high-risk mining regions with sometimes less responsible labor conditions. A second important constraint in this context is that elements such as nickel and cobalt are also needed for the production of battery electrodes, superalloys, and special steels, a situation that leverages strong market competition that will lead to massive price increases for these already expensive elements when high amounts of HEMs are manufactured from them. This applies to both primary and secondary synthesis.

HEMs belonging to the second group (Senkov alloys) face severe challenges in regard particularly to the primary synthesis of the elements containing titanium, niobium, vanadium, zirconium, and hafnium owing to their extremely high binding energy to oxygen. This circumstance makes the actual metal extraction step very costly, while many ores containing elements such as titanium, niobium, vanadium, and zirconium often have much higher metal contents than, e.g., nickel- and cobalt-containing minerals that are used for Cantor alloys. Similar arguments apply to the other high-melting refractory elements that stabilize Senkov-type HEMs into a bcc phase state. Also, the requirement to control the low oxygen partial pressure of titanium, hafnium, zirconium, niobium, vanadium, molybdenum, tungsten, and tantalum requires synthesizing and processing the materials under vacuum or very clean protective atmospheres and ensuring a very low partial pressure of oxygen [<10^−5^ torr (*83*)] and high-purity inert gas [>99.999% Ar (*84*)] at high production cost.

Secondary synthesis of Senkov HEMs will also be challenging to achieve because the corresponding scrap is already today used in existing recycling cycles. In addition, the degree of oxygen contamination in some cases is so high that the scrap can only be downcycled into oxide products, such as chipping scrap from aerospace titanium part machining. The above challenges are fundamentally associated with the immense oxygen solubility of refractory elements: In contrast to the formation of stoichiometric oxides, more than 10 at % oxygen can reside in the lattices of titanium, zirconium, and hafnium as interstitials (*85*–*87*). Without dedicated removal or purification steps, these oxygen interstitials might cause two types of core dilemmas from a physical metallurgy perspective: (i) difficulties in precisely tailoring the thermodynamic/mechanical stability of the bcc phase. Similar to the majority of beta titanium alloys (*88*, *89*), the bcc phase fields in titanium, zirconium, and hafnium-rich refractory HEMs reveal salient sensitivity to the content of interstitial oxygen and can—as a result of oxygen contamination—sometimes exhibit more complex transformation pathways and phases, e.g., the formation of the harmful omega phase (*88*, *90*, *91*), compared to oxygen-free reference materials. Likewise, the predominant plasticity micromechanisms can be unexpectedly altered by the presence of interstitial oxygen, which is not only limited to the well-documented mechanisms of screw dislocation mobility (*92*, *93*) but also broadly extended to cases of stress-assisted martensitic transformation or mechanically driven omega phase back transformation. (ii) The prevalent risk of oxygen embrittlement: This second harmful effect is due to the presence of highly mobile interstitial oxygen in the matrix, which can segregate to grain boundaries during thermomechanical processing. This type of chemical grain boundary decoration decreases the cohesive strength of the interfaces and affects their mobility. A density functional theory-based simulation has also shown that the segregation of oxygen at the grain boundary increases the electron locality in a NbMoTaW refractory HEM (*94*). This segregation leads to more significant ionic bonding characteristics, i.e., increased bonding directionality, according to Lewis’ theory of crystals (*95*, *96*), thus affecting the plastic response of the grain boundaries. The abovementioned challenges also apply to other interstitial elements, such as hydrogen, sulfur, and phosphorus, which may contaminate the final alloys and deteriorate the product toughness, ductility, and stress corrosion response. In addition to these synthesis and processing constraints, the high price of these materials will prevail or increase in the coming years due to the scarcity of elements and the growing market demand for the same elements in other products in mass-market products, such as battery electrodes, structural parts in the aerospace sector, magnets, and special steels.

These aspects suggest that the future development of HEMs should focus particularly on eliminating expensive elements as far as possible and developing HEMs that exhibit high value-added features and previously unknown property spectra, reaching far beyond what is already offered by existing and more sustainable material solutions. These materials could, for example, particularly aim at reconciling multiple functionalities and withstanding exposure to harsh operating conditions, thus becoming more sustainable by leveraging greater longevity and better performance (*27*, *35*, *94*, *97*). Along these lines, it is noteworthy that certain functional feature combinations realized in HEMs were previously thought to be mutually exclusive in conventional materials (*42*). These material design approaches offer opportunities for levering corresponding property advantages, which in a certain cases might justify the use of less sustainable elemental ingredients.

**Fig. 5.**
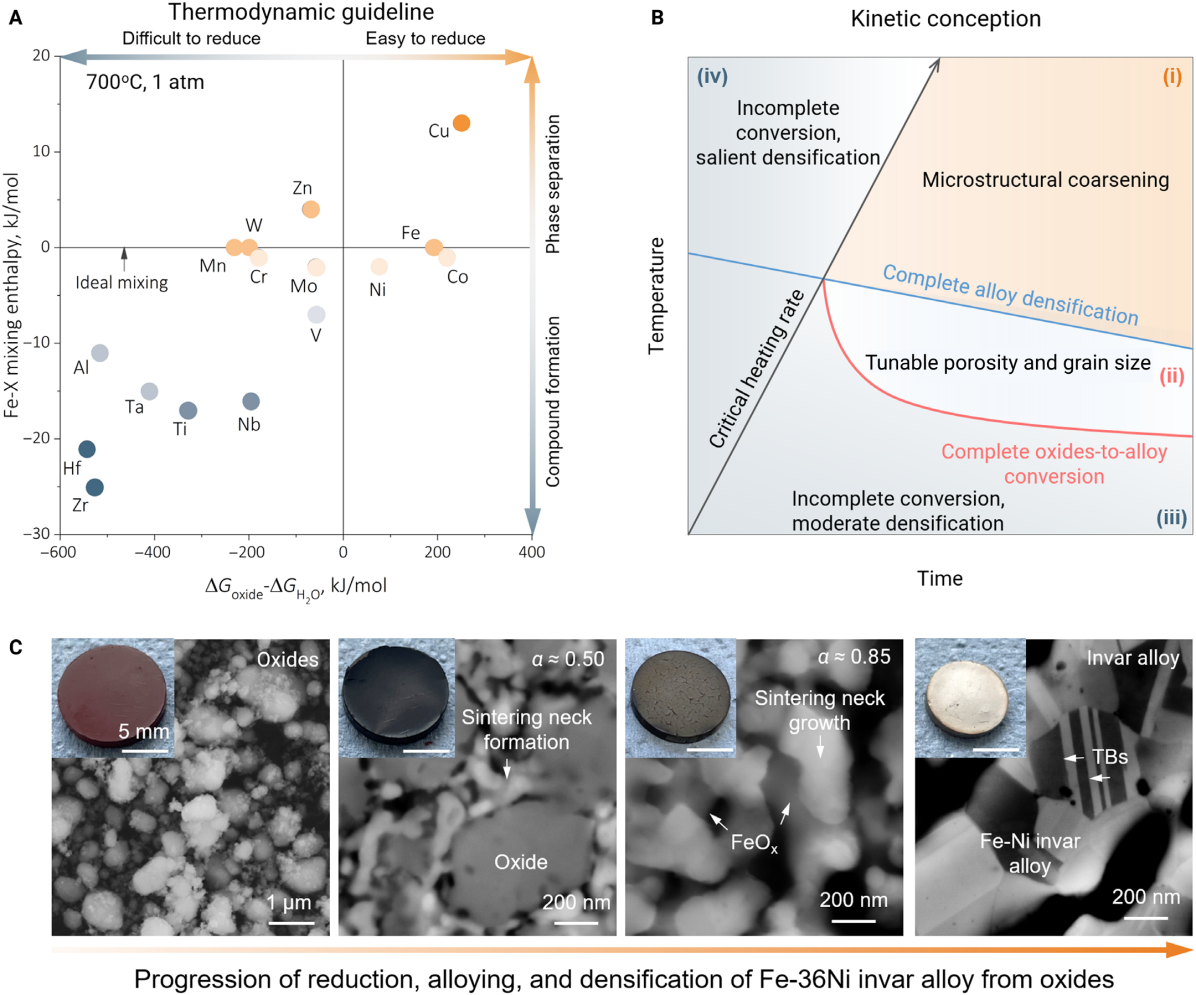
The concept of one-step synthesis of bulk HEM from oxides. (**A**) Thermodynamic design guidelines considering the bulk reducibility and substitutional alloying capability. (**B**) Kinetic conception sketching the possible microstructure states while considering the temperature, time, and conversion rate. (**C**) Microstructure evolution during the synthesis of bulk Fe-36Ni invar alloys from Fe_2_O_3_ and NiO using H_2_. Figure reproduced with permission from (*98*).

One specific possibility for the future primary synthesis of HEMs might also lie in not using high-purity elemental ingredients but instead attempting to exploit natural elemental mixtures that contain mixed oxide precursors of several transition metals (*98*). The a priori thermodynamic guidelines and an *Ashby-type* kinetic concept have recently been proposed for this approach (*98*) because the oxides of iron, copper, nickel, and cobalt exhibit, partly, comparable bulk reducibility, i.e., even at 700°C in a H_2_ atmosphere under ambient pressure (Fig. 5A). The Fe-X binary alloys are used here as an example, where X stands for the conceived substitutional alloying element(s). Two physical parameters are considered, i.e., the difference between the mixing enthalpy of oxides and water ($∆G_{\text{oxide}}-∆G_{H_{2}O}$), which indicates the bulk reducibility of the oxides in H_2_, and the mixing enthalpy between X and Fe, which shows if a solid solution can be formed or not. This guideline paves a feasible pathway to integrate metal extraction, alloying, and consolidation in one single solid-state process step with zero CO_2_ emission and suitable kinetic boundary conditions (Fig. 5B). The principle feasibility of such a sustainable one-step oxide reduction and synthesis concept has been recently validated in terms of the sustainable fabrication of Fe-36Ni [weight % (wt %)] invar alloys using a mixture of Fe_2_O_3_ and NiO under a H_2_ atmosphere, as indicated by the characteristic microstructural evolution (Fig. 5C). Oxide reduction, solid-state alloying, and densification concurrently occur, resulting in a fine-grained (average grain size of ~0.58 μm) single-phase fcc microstructure. Compared with conventional metal processing approaches, which involve fossil-based metal extraction, melting-casting, and thermomechanical processing as separate steps, this sort of sustainable oxide-to-bulk alloy operation is estimated to reduce the synthesis energy costs (~6.97 GJ/metric ton) by ~41% (*98*). Exploring further microstructure design pathways associated with such an approach is also a profound and worthwhile task to pursue; on the basis of which, the combination of grain size, phase constitution, and diverse porosity levels, e.g., even the pore topology, can be tailored via different post-reduction treatments, as outlined in the kinetic conception map (Fig. 5B).

Unlike the high-purity iron oxides currently used for blast furnaces and direct reduction-based steel production, most other naturally occurring nonferrous oxides currently used in the metallurgical sector to synthesize and extract elements are highly mixed and contaminated minerals, usually with relatively high gangue contents, making processing and extraction generally very expensive and CO_2_ intensive. As an example, nickel is primarily extracted from laterite and sulfide ores with nickel contents of approximately 1 to 2 wt %, cobalt is obtained from cobaltite and erythrite ores with cobalt contents ranging from 0.1 to 2 wt %, manganese is extracted from pyrolusite and rhodochrosite ores with manganese contents of approximately 35 to 60 wt %, and copper is sourced from chalcopyrite and malachite ores containing copper contents of only 0.5 to 3 wt %.

Another more disruptive primary synthesis approach for making HEMs may therefore lie in exploring opportunities to coextract several elements (e.g., cobalt, chromium, iron, manganese, and nickel) together from their naturally occurring oxide minerals [e.g., asbolane (*99*), chromite (*100*), hematite (*101*), pyrolusite (*102*), and josephinite (*103*)] in the same extraction cycle as premixed precursor materials to make HEM synthesis easier and at lower costs and carbon footprints. In other words, exploring unexplored HEM compositions that are closer to the metal content of mixed natural minerals might be an option. This approach does not involve first decomposing all naturally occurring minerals into single elements (e.g., CoO→Co, Cr_2_O_3_→Cr, Fe_2_O_3_/Fe_3_O_4_→Fe, MnO_2_→Mn, and NiO→Ni), which makes the process extremely carbon intensive compared with steel production but instead uses precursors from extractive metallurgy that may be closer to some of the targeted HEMs (*98*).

The secondary synthesis of HEMs refers to making HEMs from low-cost feedstock and scrap, which can be further classified as new (prime or prompt) or old (obsolete) scrap. The former is generated during the manufacturing process before being used by an end customer, whereas the latter is mixed scrap from end-of-life products (*104*). Switching from primary to secondary synthesis is often ecologically and commercially attractive (*105*, *106*). However, the availability of HEM-related scrap is very limited because HEMs are a previously unidentified grade of engineering material. Therefore, low-cost feedstock types containing a high amount of the required elements in precursor form could instead be used for the secondary synthesis of HEMs (*107*). For example, low-cost ferroalloys (*108*) serving as feedstock for iron-containing HEMs are suitable. One important advantage of using secondary synthesis to prepare HEMs is its comparatively low cost, although the availability of high-quality scrap is becoming increasingly limited because it is also cycled in other high-price mass metallic markets, such as cobalt- and nickel-based alloys, magnets, stainless steels, and special steels. A comparison of the costs of synthesizing CrCuFeMnNi HEMs via different synthesis routes revealed that the cost of secondary synthesis, i.e., the cost of using iron-nickel-chromium ferroalloys, nickel-chromium alloys, copper, manganese, and chromium scraps, is ~$1750/metric ton, which is approximately one-tenth of that of primary synthesis via pure metals (~$17,800/metric ton) (*107*). The HEM fabricated by secondary synthesis indicated an enhancement in yield strength (~230 MPa with 1 wt % of Si) compared to the one obtained via primary synthesis (~140 MPa without Si) (*107*). The yield strength was estimated using the commercial ThermoCalc software in combination with a yield strength model based on the thermodynamic equilibrium conditions for different bulk chemical compositions. The calculations were done assuming identical solidification conditions with constant grain size. Yet, further microstructural characterization and property measurements need to be conducted to confirm the simulation results.

Similar observations were made when iron-chromium, iron-manganese, and iron-nickel ferroalloys were used as raw materials for the production of the Cantor CoCrFeNiMn HEM instead of pure metals, as presented in Fig. 1B (*109*). Compared with those of primary synthesis, the costs and CO_2_ footprint were reduced by ~75 and 70%, respectively, through secondary synthesis (Fig. 2B). Another possible benefit, which needs to be further explored, is that HEMs can accommodate a wider variety of scraps and thus might be able to exploit their inherent design flexibility because of their large chemical solid solution space compared with that of conventional materials. This feature might help accommodate a higher solute content and varying solute contents from charge to charge. In addition, different types of scraps could then be mixed as feedstock materials without complex sorting or separation. This approach could reduce CO_2_ emissions by recycling scraps from conventional metal production, contributing to ~25% of total CO_2_ emissions (*110*). If scraps are too scattered and dispersed, then the CO_2_ emissions associated with collecting and using them as feedstock for secondary synthesis may be larger than those associated with conventional sustainable metallurgy.

The HEM concept also sparks the potential application of microstructural metastability engineering in alleviating some of the preceding sustainability concerns. For instance, the massive compositional space of HEMs might render the possibility of fine-tuning solid solution strengthening along with active elemental partitioning—such as manganese, nickel, and cobalt—and unlocking the well-documented TRIP effect (*12*). In this regard, alloying with cobalt scrap (where the major metallic impurities are nickel, chromium, iron, and manganese) might be of particular interest, not only because of its high cost as a raw material but also because of its ability to contribute to the bidirectional/sequential TRIP effect (*111*, *112*) and plasticity-driven microfaulting (*113*, *114*) in sustainable HEMs, both of which resemble the coherent modulated structure historically observed during the martensitic transformation of pure hexagonal close-packed cobalt (*115*–*117*). In addition, inexpensive and abundant interstitial elements that alter the stability and compositional range of the austenitic phase field can also be used in these metastable alloy design strategies.

The secondary synthesis of HEMs requires an understanding of the effects of multiple scrap-related contaminants/impurities, e.g., oxygen, nitrogen, sulfur, and phosphorus, on the microstructures and properties. For example, different types of scraps—including carbon steel (*118*), high-alloyed medium/high-Mn steel scrap materials (*119*), and stainless steel (*120*)—can supply chromium, nickel, manganese, iron, etc., for HEM manufacturing. One main challenge in the secondary synthesis of HEMs, however, is the formation of nonmetallic inclusion particles, e.g., oxides, nitrides, carbides, and sulfides, formed by the interaction between active metallic elements and impurity elements (carbon, nitrogen, sulfur, and phosphorus) compared with those in primary synthesis, i.e., metallic and nonmetallic inclusions. Nonmetallic inclusion particles can lead to deterioration of the mechanical properties of alloys. For instance, the formation of manganese-chromium-aluminum–type oxides in CoCrFeMnNi HEMs initiates the nucleation of voids during tensile deformation, thus leading to early fracture (*121*). Other aspects refer to complex and challenging metallurgical processes when HEMs are produced. Certain tramp elements, e.g., copper, which is difficult to remove and separate from the matrix because of its nobility relative to the other transition metals, accumulate below the surface oxide layers and cause hot shortness in an iron-enriched matrix during the hot-rolling process because it becomes liquid, thus causing delamination and cracking (*122*).

A third group of challenges relates to the reduced resistance of tramp element-contaminated materials when they are exposed to harsh application environments, e.g., causing corrosion and hydrogen embrittlement. For instance, the formation of MnO·Cr_2_O_3_-type oxides (*123*) in a CoCrFeMnNi HEM can initiate localized corrosion and lead to low pitting corrosion resistance. Another challenge is the shift in the chemical composition to highly contaminated feedstock and scrap. For example, HEMs fabricated from high-carbon ferrochrome scrap (*124*) can cause unwanted intergranular corrosion due to the formation of brittle M_23_C_6_-type intermetallics and chromium depletion zones. This phenomenon has not been reported in HEMs but has been identified in austenitic stainless steel (*125*). However, the additional solid solution and precipitation strengthening due to the introduction of interstitial carbon and carbides can increase the mechanical strength, so that certain trade-off considerations may apply, as is known from steel design (*126*–*128*).

Therefore, future research should focus on improving the properties of HEMs fabricated by secondary synthesis as well as refining, controlling, and distributing impurity levels to minimize their detrimental effects. For example, sulfur is a typical impurity element that can lead to thermal shock during casting and must be removed. From this perspective, one solution for purifying HEMs with high impurity levels is the conventional refining method used for structural materials, e.g., steel (*129*). For example, an ultralow-sulfur [3 to 30 parts per million (ppm)] CoCrFeMnNi HEM (*130*) was synthesized by using the complex CaO-MgO-Al_2_O_3_ oxide slag complex through induction melting with ferroalloy feedstock [sulfur ~10 to 500 ppm (*131*)]. Slag is used in such a context to dissolve sulfur and thus remove nonmetallic inclusions from the melt (*109*). Another refining method that is widely used in the steel industry (*132*) and potentially beneficial for purifying HEMs is the tuning of physical parameters, e.g., optical basicity (*133*) and viscosity (*134*), to increase the cleanliness of the melt.

The tertiary synthesis of HEMs uses dumped wastes from the mining and metal industries to produce useful HEMs. This is an additional approach for addressing the associated environmental burden by turning costly dumped waste into valuable metals (*135*). Dumped waste—including slag, ashes, dust, waste rock material, and mine tailings (*136*–*138*)—is often returned to the market in a highly chemically contaminated form, containing the elements of interest in a very dilute and mostly oxidized state. Typical dumped metallurgical industry waste types include materials such as red mud (*139*), copper slag (*109*, *140*), ferroalloy slag (*141*), and nickel laterite slag (*142*–*144*). Many of these industrial and post-consumer residues and waste materials have been dumped (for prices that can exceed the CO_2_ emission spot price by a factor of 3 to 5) or are used for landfill, an approach that is becoming increasingly restricted because of leaching effects that can contaminate soil and water (*145*). To use dumped waste as new (urban mining) raw materials to produce HEMs, different types of oxides—e.g., CaO, SiO_2_, MgO, FeO/Fe_2_O_3_/Fe_3_O_4_, and Cr_2_O_3_—need to be reduced. Although all oxides can be thermodynamically reduced by hydrogen-plasma reduction on the basis of their negative Gibbs free energy, e.g., −2.31 × 10^3^ kJ for CaO, −2.38 × 10^3^ kJ for MgO, −4.89 × 10^3^ kJ for SiO_2_, −7.84 × 10^3^ kJ for Fe_2_O_3_, and −7.54 × 10^3^ kJ for Cr_2_O_3_ (as calculated by the commercial thermodynamic software FactSage 8.3 in conjunction with the FactPS and FToxid databases at 1600°C), this process is unsustainable owing to its high energy consumption and high material loss during synthesis. Second, the techno-economic assessment must also be considered for actual applications. One successful example is the production of green steel from red mud by the hydrogen-plasma reduction method (*68*). However, assessing other types of dumped waste materials is also worth investigating. Third, further questions associated with collecting and sorting reduced metals from waste and mixing them as raw materials for HEM production require attention. Last, the preconditions for reusing dumped tailings and industry waste have become feasible from commercial and sustainable perspectives. This process will provide a growing market for more expensive materials, e.g., HEMs. In addition, more robust metallurgy methods, such as pyrometallurgy or hydrometallurgy, are needed to extract metallic elements, and industry residues urgently need to be used as untapped urban mining feedstocks to increase the future sustainability of HEM production.

### Indirect sustainability aspects: Chemical, microstructural, and processing strategies that leverage sustainability through the use of lean HEMs

**Fig. 6.**
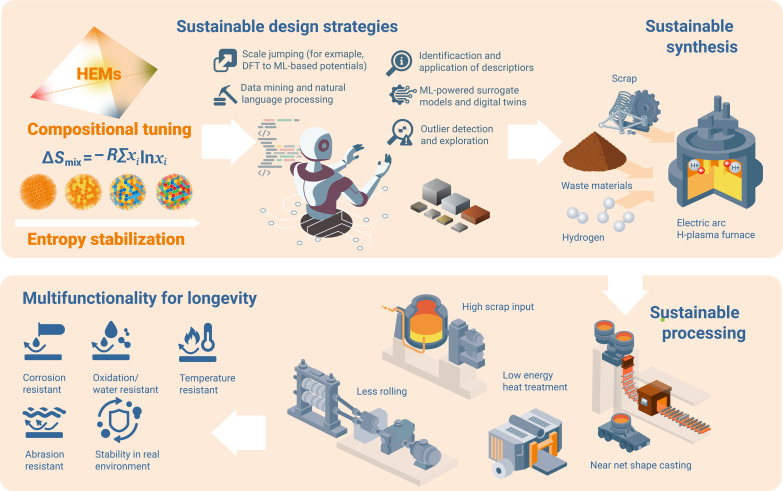
Sustainable design and manufacturing strategies. Different measures have to be taken into account in the design, synthesis, and processing of mass-produced HEMs to reduce energy consumption and CO_2_ emissions and ensure multifunctionality and longevity.

HEMs must be designed, synthesized, and processed to compete with conventional advanced materials in terms of properties, sustainability, responsible production, pricing, and recyclability. These aspects are sometimes overlooked in the field of material design, and particularly, the constraints set by sustainability become more severe by the hour. The design of HEMs includes exploring alloy variants with lean chemical compositions, coping with variable feedstock quality, and specifically with variable impurity contents. All these aspects must be considered as additional parameters in the thermodynamic and kinetic design when tailoring the complex microstructures of these materials (Fig. 6). More specifically, the core idea of the HEM concept, namely, entropy-based high targeted matrix solubility, can probably be exploited to better cope with moderately varying chemical compositions between the scrap charges for digesting variable scrap types without creating abrupt property changes. Another aspect that seems to offer a promising avenue for finding a place for HEMs on the map of industrially applicable materials lies in the design of compositionally lean and, at the same time, multifunctional materials that might, in a few cases, be comparable to or even surpass the properties of established commercial alloys. Materials with multifunctional property profiles are important because key applications for emerging real-world materials cannot be served by a solitary property dimension alone but often need to satisfy a spectrum of conflicting properties that are difficult to reconcile in a single alloy.

An example are mechanically robust soft magnetic HEMs because enhancing mechanical strength and strain hardening typically requires lattice defects such as dislocations and interfaces, which increase coercivity and the associated hysteresis losses (*146*, *147*). Another promising option is the development of improved Cantor-related HEM catalysts, which can potentially compete with or surpass existing iridium- and platinum-based catalysts for certain reactions (*148*, *149*). We summarize some opportunities where HEMs might provide better performance than established materials to leverage altogether higher system sustainability in Table 1. Here, we envisage the design of HEMs as sustainable alternatives to existing materials with currently high embodied energy, CO_2_ footprint, cost, and supply risk (*150*–*152*), such as established alloys made from cobalt (*153*), RE elements (*154*), or platinum-group elements (*155*).

Following these ideas, design opportunities for sustainable HEMs are based on large solid solution ranges with potential multifunctional properties. For example, the binder phase (most typically pure cobalt) in hard metals (e.g., WC-Co) requires a multifunctional property profile, including wettability during liquid sintering, high-temperature stability, good oxidation resistance, corrosion resistance, and fracture toughness (*32*). Compositionally lean HEMs with high configurational entropy, unchartered phase states, and sluggish diffusion can help stabilize the binder phase against property degradation over a wide temperature range (*156*). For instance, the costs and the CO_2_ footprint of the WC-CoCrFeNi HEM are reduced to ~60 and ~75%, respectively, compared to the WC-Co reference material. In addition, the WC-CoCrFeNi HEM shows enhanced resistance against plastic deformation during the cutting test, i.e., the plastic deformation is reduced to only ~15% of that of commercial WC-Co at a turning speed of 150 mm/min (*153*).

Another research direction for compositionally lean HEMs pertains to permanent magnets. Exploiting the compositional complexity and entropy engineering of transition-metal sites in RE permanent magnets can enhance magnetic moments by stabilizing ferromagnetic ordering and electron-spin coupling at the Fermi level, thus improving the performance by forming stronger ferromagnetic phases while reducing the raw material cost for improved sustainability. For example, substituting cobalt with the more sustainable element iron in the SmCo system can simultaneously increase the magnetization and the Curie temperature, a feature that is essential when hard magnets are used under high-temperature conditions such as those encountered in fast rotation electrical engines. More specifically, the magnetization increases from 7.8 to 10.6 μ_B_ with increasing Fe concentration *x *from 0 to 0.8, and the Curie temperature increases from 890 to 1357 K in the Sm(Co_1−*x*_Fe*_x_*)_5_ system. This is attributed to the strong exchange interaction of the Fe─Co coupling, according to density functional theory calculations (*157*). Similarly, the effects of the addition of low-cost elements—including iron, titanium, nickel, zirconium, hafnium, manganese, and chromium—on the magnetic properties at transition-metal sites have been screened in SmCo permanent magnets (*158*) and extended to other material systems (*159*–*161*). For example, substituting cobalt ($50,000/metric ton) with the relatively lower cost element copper ($10,000/metric ton) in SmCo_5−*x*_Cu*_x_* increases the coercivity from 4.5 kOe (SmCo_5_) to 5.97 kOe (SmCo_3_Cu_2_) and 6.99 kOe (SmCo_4_Cu) due to the increased anisotropy energy (*162*). The compositional-complexity alloy design approach has thus reduced the total cost of the material by 5% at increased saturation remanence.

These compositionally lean and continuous HEM design examples also provide access to a wide range of complex microstructures, kinetics, nonequilibrium phase transformations, chemical ordering, and decoration phenomena. The resulting microstructures can differ from those in conventional alloys and provide additional opportunities for property shaping by introducing microstructural features across different length scales via thermomechanical processing. Thus, specific properties can be achieved by tuning the chemical composition, size, dispersion, and morphology of the microstructural defects and not the entire bulk material. These microstructural defects with one or the combination of several well-tuned parameters might trap unwanted impurities beyond their equilibrium solubility limit, leveraging a protective means against the formation of unwanted inclusions and property degradation when tapping into sustainable low-purity feedstock. A specific example along these lines of impurity-tolerant microstructures of HEMs is the eutectic lamellae concept (*75*, *163*, *164*). When rapid solidification is used to increase the interface density, HEMs with fine eutectic lamellae can be an approach for the sustainable production of strong and tough alloys. More specifically, eutectic HEMs solidify at a fixed solidification point where a mixture of two massive solid solution phases coexists with the liquid. As the solubilities of the two phases are different, the phase with higher mutual solubilities for impurities usually has a higher segregation trend for lattice defects and a higher chance of trapping impurities via rapid solidification. The high growth velocity at the liquid-solid interface refines the lamellar spacing, thus increasing the interface density (*165*–*167*). For such a design strategy, an entropy-driven and multiphase design can open opportunities for a more sustainable chemical and microstructural design that does not encompass the entire bulk material.

Given that HEMs use a high fraction of low-price feedstock, scrap, and dumped waste materials, reaching similar or enhanced properties will remain challenging compared with reference materials that are high-cost, high-supply risk, and high-energy feedstock materials. Therefore, a certain inevitable loss in material performance for high gains in sustainability might become an additional and, in some cases, acceptable metric for HEM design. This could pay off, particularly in cases where high benefits in specific application scenarios of HEMs do not require top performance for only one singular feature but rather a multifunctional property spectrum, including a sustainable production protocol. In other words, it might be worth exploring applications in which it might make sense (in light of a properly conducted life cycle assessment) to trade the peak performance of an expensive and less sustainable material for a less performant but substantially more sustainable material. This idea indicates that the introduction of corresponding mathematical optimization assessments might help to calculate and evaluate the trade-off between price advantage, CO_2_ emission mitigation, and loss in functionality and/or performance of (sustainable) HEMs, thus going beyond standards established today by conventional life cycle assessments. In this context, machine learning–based methods can help predict and calculate these features, as shown in Table 2.

**Table 2.** Simplified example of possible workflows based on the use of large language models in connection with downstream thermodynamic, kinetic, life cycle assessment and cost modeling tools. Notably, the prompts and results have been markedly shortened in this table, and the full return items have been much more detailed.

Incentive	Example prompts for interrogating a large language model	Types of results obtained	Submission of these results to more detailed and quality-controlled (hallucination-free) calculations by conventional modeling methods
Get market survey on metal scrap prices	Screen suited trading platforms for metal scrap prices.	Reliable trading platforms for highly alloyed scraps.	Use quality controlled trading platforms for scrap prices and check further metrics such as availability, quantity, quality, contamination. Price volatility, orgin of material, transport costs, etc.
	Focus on low price postconsumer scrap.		
What are available low price and sustainable feedstock options for HEMs (here for FeNiCoMnCr HEM as an example)	Find scrap that is high in Fe, Ni, Mn, Co, Cr content.	Types of scrap that are available on international scrap markets.	Narrow down on prices for the specific scrap sorts needed to make the target HEM.
Develop basis for a first cost estimate on the feedstock side	Specific prices.	Examples of prices ($/metric ton): Stainless steel scrap (304 of 316): $900–1200, automotive shredded scrap: $200–$250, nickel alloy scrap: $3000–5000.	Do exact cost calculations including transport, melting points, specific contamination information, processing etc.
Some first data required for a life cycle assesment	CO_2_ footprint for these scrap types and comparison to CO_2_ footprint data when making the same materials from minerals.	CO_2_ footprint data: Stainless steel scrap (304 of 316): recycling: 0.7–1.0 metric tons CO_2_ per metric of metal. Primary production: 4.0–6.0 metric ton CO_2_ per metric ton of stainless steel produced from raw materials (Fe, Ni, Cr, etc.). Automotive shredded scrap (ASR): Recycling: 0.5–1.0 metric ton CO_2_ per metric ton of metal. Primary production: 2.0–3.5 metric ton CO_2_ per metric ton of metal. Nickel alloy scrap: Recycling: 1.5–2.0 metric ton CO_2_ per metric ton of nickel alloy. Primary production: 8.0–12 metric ton CO_2_ per metric ton of nickel alloy.	Make specific CO_2_ calculations considering details of the HEM composition, transport, processing, etc., using, for example established LCA software tools.
Overlap optimal cost model with target material	Identify best mix of low price scrap sources to make the target alloy.	Stainless steel scrap: $900–$1200/metric ton: 30% of mass. ASR: $200–$250/metric ton: 10–15% of mass. Nickel alloy scrap: $3000–$5000/metric ton: 10–15% of mass.	Make specific CO_2_ calculations considering details of the HEM composition, transport, processing, etc. using for example established LCA software tools.
Add to this as a constraint the optimal CO_2_ footprint	Combine feedstock costs and CO_2_ footprint.	1. Fe and Mn: Primarily from ASR to keep both costs and CO_2_ emissions low.	Combine composition with CO_2_ and property calculations.
		2. Cr: From a combination of stainless steel scrap and ASR.	
		3. Ni: Mainly from stainless steel scrap, minorly from nickel alloy scrap.	
		4. Co: Primarily sourced from nickel alloy scrap, kept to a minimum due to its high CO_2_ footprint.	
Identify options for composition and property loss traded for gain in sustainability	Combine all of the above results to make specific alloy variant suggestions.	Use scrap with reduced purity, lower Ni and Co content, highest sustainability gain comes through reducing the fraction of nickel and cobalt alloy scrap, increased use of lower-cost and lower CO_2_ carrying elements such as Fe and Mn, sustainability gained by maximized use of scrap and minimized use of sweeting primary material	Use thermodynamic and kinetic models to do the actual phase diagram and cooling simulations. Use microstructure-property simulations to relate the results to property losses per gain in sustainability obtained from LCAs.

As an example, large language models can already today be used for these purposes. We can leverage these methods, for instance, as tools for developing a decision framework considering various sustainability metrics and potential application requirement criteria, such as the CO_2_ footprint, environmental, social, and governmental (ESG) impact associated with mining, and multifunctional property spectra, as outlined above. To demonstrate this approach, the equiatomic FeNiCoMnCr HEM is used here as an example of how a rough workflow can be envisaged to provide a future platform approach for the development of more responsibly produced HEMs. This can also be transferred to many other types of materials, feedstocks, and processes. These comparative analysis can improve the efficiency and speed of sustainable HEM design under such multiconstraint conditions through large language model support and data-driven correlation studies (*168*, *169*).

### Directions for future work

We outlined approaches and strategies for developing sustainable HEMs and discussed sustainable synthesis and manufacturing and less energy- and CO_2_-intensive design strategies.

Some HEMs exhibit impressive structural and functional behavior due to their multi-principal-element composition. These characteristics provide them with properties potentially suitable for structural components in extreme environments (cryogenic, hot, irradiation, corrosive, etc.), magnetic components, energy conversion, catalysis, hydrogen economy, etc. However, HEMs are generally burdened by cost-, energy-, and carbon-intensive extraction, synthesis, and manufacturing processes. For some HEMs, the CO_2_ footprint is up to 50 times larger than that of conventional materials. In addition, recycling and reusing HEMs are challenging because their design relies on high fractions of expensive and limited elements in massive solid solutions for which matching scraps are unavailable in sufficient quantities.

We therefore summarized the fundamental sustainability issues of HEMs and discussed specific aspects and mitigation strategies associated with manufacturing, material design, properties, and recycling. Possible mitigation strategies include the use of feedstocks with lower energy and carbon footprints, sustainable primary synthesis pathways sourced from mixed minerals, deviation from the equimolar alloying rule, and the use of scraps and dumped waste materials for secondary and tertiary synthesis. Specifically, the high solubility, compositional flexibility, and chemical robustness of HEMs might provide avenues for using greater fractions of mixed and contaminated scraps and dumped waste than are admissible for manufacturing conventional materials. Furthermore, we discussed thermodynamic and kinetic design strategies to develop good material properties with high-impurity tolerance and variable compositions.
